# Endocrine Toxicities of Antineoplastic Therapy: The Adrenal Topic

**DOI:** 10.3390/cancers14030593

**Published:** 2022-01-25

**Authors:** Agnese Barnabei, Paola Senes, Alessandro Scoppola, Alfonsina Chiefari, Giovanni Maria Iannantuono, Marialuisa Appetecchia, Francesco Torino

**Affiliations:** 1 Endocrinology Unit, P.O. S. Spirito in Sassia, ASL Roma 1, Lungotevere in Sassia 1, 00193 Rome, Italy; agnese.barnabei@aslroma1.it (A.B.); alessandro.scoppola@aslroma1.it (A.S.); 2 Internal Medicine Unit, P.O. S. Spirito in Sassia, ASL Roma 1, Lungotevere in Sassia 1, 00193 Rome, Italy; paola.senes@aslroma1.it; 3 Oncological Endocrinology Unit, IRCCS Regina Elena National Cancer Institute, Via Elio Chianesi 53, 00144 Rome, Italy; alfonsina.chiefari@ifo.gov.it (A.C.); marialuisa.appetecchia@ifo.gov.it (M.A.); 4 Medical Oncology Unit, Department of Systems Medicine, University of Rome Tor Vergata, Via Montpellier 1, 00133 Rome, Italy; gmiannantuono@gmail.com

**Keywords:** primary adrenal insufficiency, endocrine toxicity, immune checkpoint inhibitors, anticancer drugs

## Abstract

**Simple Summary:**

Primary adrenal insufficiency (PAI) induced by anticancer drugs is a rare occurrence. However, with the expanding use of immune checkpoint inhibitors (ICIs), an increasing number of patients affected by ICI-induced PAI are expected. As a result, oncologists should be aware of the risk of PAI in patients on ICIs (and other anticancer agents) and the related clinical presentations to diagnose this condition early, provide the appropriate treatment, and timely involve endocrinologists in the management of those patients. In parallel, patients on these medications, together with their caregivers and relatives, should receive detailed information about PAI risk and be trained to act appropriately at the onset of alarm symptoms. Large collaborative trials are needed to develop appropriate tests to assess the personal risk of drug induced PAI better and improve its early diagnosis in cancer patients and patients affected by other forms of PAI.

**Abstract:**

Immune checkpoint inhibitors (ICIs) have improved survival in patients affected by several solid tumours at the cost of new autoimmune adverse events. Endocrine toxicity is frequently reported in patients treated with these agents, mainly as thyroid dysfunction and hypophysitis. Primary adrenal insufficiency is reported in 1–2% of patients receiving a single ICI, but its rate is approximately 5% in patients treated with a combination of two ICIs. The clinical presentation of adrenal insufficiency may be insidious due to symptoms that are not specific. The same symptoms in cancer patients are frequently multifactorial, rendering the early diagnosis of adrenal insufficiency challenging in this group of patients. As adrenal insufficiency can be fatal if not rapidly diagnosed and treated, oncologists should be aware of its clinical presentations to timely involve endocrinologists to offer patients the appropriate management. In parallel, it is essential to educate patients, their caregivers, and relatives, providing them with detailed information about the risk of adrenal insufficiency and how to manage alarming symptoms at their onset. Finally, large collaborative trials are needed to develop appropriate tests to assess better the personal risk of drug-induced adrenal insufficiency and its early diagnosis and treatment, not only in cancer patients.

## 1. Introduction

The adrenal gland comprises two distinct anatomical and functional parts: the cortex and the medulla. The cortex produces glucocorticoids (primarily cortisol), mineralocorticoids (primarily aldosterone), and androgens (primarily dehydroepiandrosterone and androstenedione). Glucocorticoids affect immunity, metabolism, and cognition, including pleiotropic effects on the immune system. Mineralocorticoids regulate electrolyte transport across epithelial surfaces, particularly renal conservation of sodium in exchange for potassium. The adrenal medulla mainly secretes catecholamines (mainly epinephrine and lesser amounts of norepinephrine), playing a central role in the sympathetic nervous system, and are responsible for the response to “acute stress” [1,2].

Adrenal insufficiency (AI) derives from decreased glucocorticoids and/or mineralocorticoids and adrenal androgens production. AI may be life-threatening and is commonly classified as primary, secondary, or tertiary. Primary adrenal insufficiency (PAI), also named Addison disease, originates from an injury inherent in the adrenal gland itself. Secondary adrenal insufficiency (SAI) results from damage to the pituitary gland, leading to a decreased secretion of adrenocorticotrophin hormone (ACTH), while tertiary adrenal insufficiency (TAI) arises from a reduced release of corticotrophin-releasing hormone (CRH) from the hypothalamus. Many conditions may trigger PAI (Table 1), including drugs [1,2]. In the present paper, we focused on PAI induced by anticancer drugs.

## 2. PAI Epidemiology and Aetiology

PAI is a rare condition, with a prevalence of 10–20 patients per 100,000 [3]. PAI peak of incidence is in the third decade of life. It is more frequently diagnosed in women than men, with higher frequencies in individuals affected by other autoimmune disorders (i.e., type 1 diabetes mellitus) [4,5,6,7,8]. In the past, tuberculosis was one of the predominant causes of PAI and remains the leading cause of PAI in regions with a high prevalence of tuberculosis, in addition to other infectious diseases such as HIV infection, and opportunistic infections (e.g., cytomegalovirus) [9,10,11,12]. Nowadays, autoimmune PAI and congenital adrenal hyperplasia are emerging as the prominent causes of PAI [13,14,15]. Autoimmune Addison disease accounts for approximately 90% of non-genetic cases of PAI in industrialized countries [16]. In Addison’s disease, lymphocytes infiltrate the adrenal parenchyma leading to adrenal fibrosis and atrophy. Antibodies directed against 21-hydroxylase are specific to autoimmune adrenalitis. Local infection by the herpes simplex virus 1, cytomegalovirus, and adenovirus are suggested as trigger factors of PAI. Moreover, some variants of genes implicated in adaptive or innate immunity affecting regulatory T-cell function seem to increase the risk of autoimmune adrenalitis [17]. However, the role of viruses and gene variants in the onset of PAI remains to be fully elucidated. Other causes of adrenal destruction and dysfunction include bilateral adrenal metastases, infections, infiltrative diseases, haemorrhage, surgery, infarction, and medications (Table 1) [18,19]. 

## 3. Clinical Aspects of PAI

AI may manifest as either an acute or chronic condition. The acute form often presents as a critically ill state (adrenal crisis), while the chronic ones can be insidious. In adrenal crisis, dehydration, hypotension, or shock are key symptoms (especially in PAI). However, anorexia, nausea, vomiting, abdominal pain, weakness, fatigue, lethargy, fever, confusion may be present. Hyponatremia, hyperkalaemia, hypercalcemia, hypoglycaemia, or eosinophilia may also occur.

In chronic AI, patients mainly complain of fatigue (≈90%), weight loss (≈70%), nausea, vomiting, abdominal pain (≈55%), and muscle and joint pain (≈38%). Moreover, hypotension, salt craving, hyponatremia, hyperkalaemia, hypercalcemia, and hyperchloremic acidosis, mild normocytic anaemia, lymphocytosis, eosinophilia may coexist [20]. Androgen deficiency may result in low libido and weakness, and fatigue. A distinctive feature of chronic PAI is melanoderma [20]. Notably, in patients with SAI or TAI, mineralocorticoid function is preserved, as mineralocorticoid levels are regulated by the renin-angiotensin system that is independent of hypothalamic or pituitary control. 

In particular, the chronic form of AI may be overlooked or misdiagnosed. Moreover, the decreasing or suppressed adrenal function may be undetected until a stressful condition (i.e., illness) leads to adrenal crisis. Remarkably, chronic AI may be insidious in cancer patients since nonspecific symptoms may frequently be due to cancer and anticancer treatments. Therefore, a high level of suspicion for this condition is required by clinicians to reach an early diagnosis and provide patients with rapid and appropriate treatment.

## 4. Diagnosis of Adrenal Insufficiency

Laboratory tests are necessary to confirm the clinical suspect of AI showing decreased levels of serum cortisol and a reduced response of cortisol secretion at the Cosyntropin stimulation test (250 mcg i.v.) [21] (Table 2). Notably, an early morning low serum cortisol level <3 mcg/dL strongly suggests AI [22,23,24]. High plasma ACTH levels distinguish PAI from the other forms of AI (SAI or TAI). Moreover, in patients with PAI, plasma renin activity (or renin concentration) is increased. This alteration could be precedent to reducing cortisol levels and could identify a preclinical phase of the disease. Hyperkalaemia, hyponatremia and hypoglycaemia are often present in PAI [21]. Remarkably, the ACTH stimulation test can only confirm AI, while only the assessment of basal ACTH before injection of Cosyntropin may help distinguish PAI from SAI/TAI. However, it should also be considered that morning cortisol assessment and ACTH stimulation test may not be informative in some conditions, such as in patients treated with oestrogens, nephrotic syndrome, inappropriate Cosyntropin dilution, acute ACTH deficiency (e.g., hypophysitis acute phase, brain surgery or trauma), critical illness or when tests are not performed at the right time (8.00 A.M.). The diagnosis of PAI is moreover complicated as the most common cause of the condition, autoimmune adrenalitis, usually worsens over many months or years. In a study on patients with increased levels of adrenal antibodies in the absence of overt AI, the disease may develop in the next three to five years, suggesting that PAI may evolve throughout four stages (Table 3) [25,26,27]. Thus, when the patient has developed low serum cortisol concentrations, adrenal destruction may be considered complete. Notably, in patients who are on treatment with glucocorticoids (e.g., dexamethasone), the diagnosis of primary AI is difficult and often misleading. In these patients, the suppression of the hypothalamic-pituitary axis induced by glucocorticoids may hide AI. The entity of suppression may vary according to the potency of the glucocorticoid agent used, its half-life (short, intermediate, prolonged), concomitant drugs interfering with glucocorticoid metabolism, systemic/local delivery, daily/alternate days of administration, split/night dosing, duration of the treatment, cumulative glucocorticoid dose, and individual susceptibility. In patients under glucocorticoids, the diagnosis of AI may be obtained with the ACTH stimulation test once the dose of prednisone (or equivalent) has been reduced to 5 mg or less. If glucocorticoid-induced suppression of the hypothalamic-pituitary axis is suspected, the ACTH stimulation test should be performed at the suspension of glucocorticoids, in cases of intolerable glucocorticoid-related adverse events, or when major surgery is needed.

An abdominal CT scan should be performed to detect enlarged adrenal glands or adrenal calcification, suggesting an infectious, haemorrhagic, or metastatic cause. Magnetic resonance imaging and FDG-PET scan may be helpful as second-level exams in the differential diagnosis of adrenal masses [28,29]. However, radiological examinations may not be necessary in all cases of suspected adrenal insufficiency, e.g., when recent scans are available, such as in cancer patients who frequently undergo staging procedures. On the contrary, timely starting glucocorticoid therapy is vital while performing a further radiological assessment.

Antiphospholipid antibodies can be helpful when bleeding is present in a patient not receiving anticoagulants [30]. The presence of autoimmune disorders can sustain the diagnosis of autoimmune insufficiency. The assessment of serum antibodies against 21-hydroxylase (P450c21) is suggested as a promising approach in diagnosing PAI, but it is not currently integrated into clinical practice [31,32]. 

## 5. Drug-Induced Adrenal Insufficiency

Several drugs may alter adrenal function. The mechanism(s) leading to adrenal toxicity are different according to the drug’s type. Some agents induce PAI by inhibiting cortisol synthesis, accelerating cortisol metabolism, or inducing hepatic mixed-function oxygenase enzymes. Others primarily suppress the corticotropin-releasing hormone (CRH) or ACTH production or potentiate glucocorticoid effects by decreasing its metabolism leading to SAI or TAI. Agents that inhibit cortisol biosynthesis include anaesthetic–sedative drugs, such as etomidate, the antimycotic ketoconazole and fluconazole, metyrapone, and the antiparasitic drug suramin (in the past also tested as anticancer agent) [1]. Notably, these drugs may cause symptomatic AI only in patients with impaired pituitary or adrenal reserve due to an incomplete adrenal enzyme inhibition and a compensative increase in adrenocorticotropic hormone (ACTH) secretion, counterbalancing the pharmacologic blockade. Rifampin, phenytoin, and barbiturates (as well as most synthetic glucocorticoids) accelerate the metabolism of cortisol by inducing hepatic oxygenase enzymes, leading to an adrenal crisis in patients with partial pituitary or adrenal reserve or in those who are on glucocorticoid replacement therapy due to AI [1,33]. Mitotane is the only agent exerting an adrenocorticolytic action. However, it also increases the metabolism of synthetic steroids, such as hydrocortisone, dexamethasone, and fludrocortisone [1,33]. Notably, mitotane can cause adrenal insufficiency in patients with adrenal carcinoma who are on treatment with those drugs.

## 6. Drug-Induced Adrenal Insufficiency in Cancer Patients

Anticancer drugs infrequently affect adrenal function, except for mitotane. Sporadically, AI is diagnosed in cancer patients treated with interferons, interleukins, megestrol acetate, aminoglutethimide, suramin (aminoglutethimide and suramin are no more in clinical use), and targeted agents, such as tyrosine kinase inhibitors (TKIs). Other drugs frequently used in cancer patients may cause AI, such as corticosteroids and opioids. These agents primarily suppress the corticotropin-releasing hormone (CRH) or ACTH production, causing SAI or TAI.

Recently, immune checkpoint inhibitors (ICIs) have emerged as anticancer drugs electively provoking endocrine toxicity, including AI.

## 7. ICI-Induced Adrenal Insufficiency

ICIs are monoclonal antibodies (mAbs) that act by blocking key regulatory signals that negatively modulate immune responses. In the tumour microenvironment ICIs overcome immune suppression, thus allowing tumour-reactive T cells to restore an effective anticancer response. Two major ICIs’ classes are clinically available: those targeting the cytotoxic T lymphocyte antigen-4 (CTLA-4) (anti-CTLA-4 mAbs) and those targeting the programmed cell death protein 1 (PD-1) and its ligand-1 (programmed death-ligand 1, PD-L1; anti-PD-L1 mAbs) [34].

ICIs have been approved to treat several advanced solid and hematologic malignancies, and more recently, as adjuvant therapy for patients affected by high-risk melanoma and urothelial carcinoma, for those affected by non-small cell lung cancer, oesophageal and gastroesophageal junction carcinoma. Moreover, studies of peri-operative efficacy of the checkpoint blockade are ongoing, and several trials are evaluating novel monoclonal antibodies or testing approved ICIs in combination with other treatment modalities, including chemotherapies or targeted agents [35].

Although ICIs have a good toxicity profile, patients may frequently experience autoimmune/autoinflammatory adverse events, commonly called immune-related AEs (irAEs). irAEs are inherently related to immunological mechanisms of action of ICIs and may potentially affect all organs, with the skin, gastrointestinal apparatus, and endocrine systems being mainly affected [36]. Hypophysitis, thyroid dysfunction, type 1 diabetes mellitus (T1DM), central diabetes insipidus, and PAI are ICI-induced endocrine irAEs [37,38]. Causative and risk factors eliciting the endocrine system’s involvement (and other organs) in ICI-related toxicity are currently unknown. It is now clear that the prevalent damage to an endocrine gland depends on the type of the ICI. Hypophysitis is mainly associated with anti-CTLA4 mAbs (3.9–8.1%), whereas thyroid dysfunction is predominantly associated with anti-PD-1/PD-L1 mAbs (6.4–9.8%) [37,39]. Moreover, the risk and severity of endocrine toxicity differ in regard to whether a single ICI or a combination of ICIs is used. Indeed, the combination of anti-CTLA4 and anti-PD-1 mAbs is associated with a higher incidence of endocrinopathies (hypothyroidism: 10.2–16.4%; hyperthyroidism: 9.4–10.4%; hypophysitis: 8.8–10.5%) than the individual use [37,39,40].

**Figure 1:**
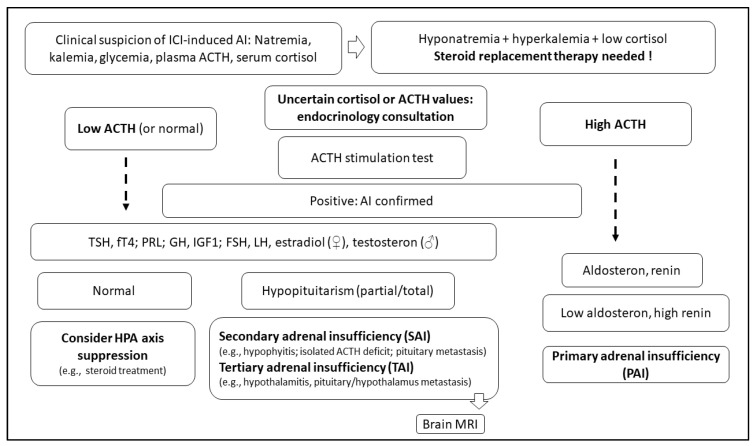
A practical algorithm to diagnose different forms of adrenal insufficiency (AI) in patients on treatment with immune checkpoint inhibitors (ICI).

AI induced by ICI is prevalently secondary, resulting from either an anterior hypophysitis or selective damage of ACTH-producing cells in the pituitary (Figure 1). One case of TAI has been reported as the consequence of the unique case of hypothalamitis induced by atezolizumab, an anti-PD-L1 mAbs [41]. ICI-induced PAI is reported in 1–2% of patients treated with anti-PD-(L)1 or CTLA-4 therapies and is estimated to occur at a rate of 5% in patients treated with combination ICIs [39,40,42]. 

However, Grouthier et al. published in 2020 the results of a descriptive analysis of Vigibase, the World Health Organization’s pharmacovigilance database of individual case safety reports. A total of 50,108 ICI-irAEs were reported from 2008 to 2018, and 451 (0.9%) cases of PAI were identified [43]. Among the subjects with PAI, 45 (0.09%) were classified as “definite PAI” and 406 (0.81%) as “possible PAI.” Patients were prevalently male (58.1%) with a median age of 66 years (range 30–95), and the main underlying malignancies were melanoma (41.2%) and lung cancer (28.6%). Most patients were treated with a single ICI (ipilimumab, nivolumab or pembrolizumab in 23.6%, 44.3%, and 11.7% of cases, respectively, and atezolizumab, durvalumab or avelumab in 1.6%, 0.7% and 0.2%of cases, respectively), while 17.9% of patients received an ICI combination therapy. The median time at onset of PAI was 120 days, with a range of 6–576 days [43,44]. Significant morbidity was associated with ICI-induced PAI, and it was classified as severe in more the 90% of cases, with a mortality rate of 7.3%. Interestingly, fatality rates were similar either for patients treated with ICI-combination therapy or monotherapy. Relevant differences were not found between the “definite” vs. “possible” PAI group in terms of clinical or demographical characteristics and outcomes [43]. 

Additionally, in a recent systematic review of the literature on cases diagnosed with PAI induced by ICI, Shi et al. [45] reported that 60% of the 15 patients included in the review showed other endocrine toxicities such as thyroid dysfunction in 66.7% of cases, T1DM in 44.4% and hypophysitis in 33.3%, with two patients who developed three concomitant endocrinopathies and one patient four endocrinopathies. 

Malaise, fatigue, nausea, light-headedness, loss of appetite, and weight loss are the most common symptoms of ICI-induced SAI’s clinical presentation, similar to that of secondary hypocortisolism. Nevertheless, secondary hypocortisolism is more frequently associated with hypotension and adrenal crisis due to the combination of severe glucocorticoid and mineralocorticoid deficiency [45,46,47,48]. Decreased morning cortisol levels and elevated ACTH levels are usually detected at laboratory testing. However, morning cortisol and ACTH levels are not always conclusive; therefore, an ACTH stimulation test (i.e., Cosyntropin stimulation test) (Table 2) may be needed [35]. In addition, metabolic acidosis and hyperkalaemia may also be detected if mineralocorticoid-producing cells are affected.

**Figure 2:**
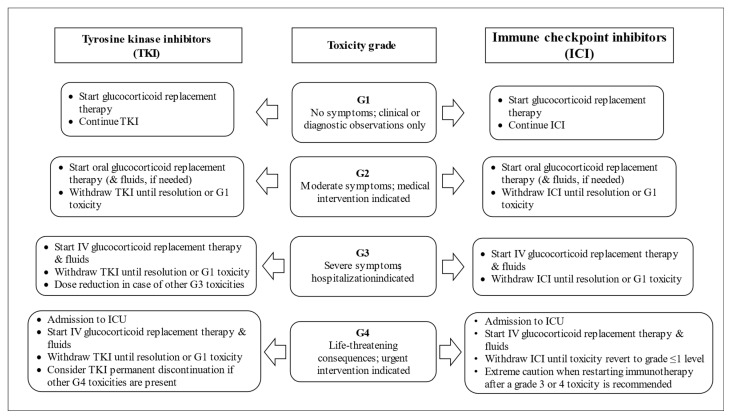
A flow diagram for managing patients diagnosed with adrenal insufficiency induced by anticancer drugs (Toxicity graded according to the current CTCAE).

Severe AI, whether primary or secondary, is a life-threatening condition. Therefore, it is mandatory to start the treatment promptly, even in the absence of confirmatory testing or subtype classification (Figure 1) [46]. As for SAI due to hypopituitarism, the therapy of PAI requires glucocorticoid replacement and might include mineralocorticoid supplementation [21,35,46,49,50,51,52,53,54,55]. As ICI-induced PAI is not a common irAE and may be vague in its clinical presentation; close monitoring of patients is necessary due to the risk of adrenal crisis. In general, patients diagnosed with ICI-induced PAI can continue their ICI therapy after recovering from acute symptoms and dysfunctions, as in cases of ICI-induced hypophysitis (Figure 2) [35,49,50,51,52,53,54,55].

## 8. TKI-Induced Adrenal Insufficiency

TKIs are small molecules that dramatically improve survival and quality of life in patients affected by various solid and blood cancers. TKIs target key tyrosine kinases implicated in tumorigenesis and progression and exert their anticancer activity by inhibiting phosphorylation of tyrosine residues of their substrates. Several TKIs with single or multiple targets, including EGFR, ALK, ROS1, HER2, NTRK, VEGFR, RET, MET, MEK, FGFR, PDGFR, and KIT, are currently available. TKIs are considered less toxic compared with cytotoxic agents, but their adverse effects can negatively affect patients’ quality of life, not rarely leading to drug discontinuation. The most common TKI-induced AEs include fatigue, decreased appetite, diarrhoea, nausea and vomiting, weight loss, hypertension, and skin reactions. In addition, several endocrine-related adverse effects have been described with the use of TKIs, such as hypothyroidism, altered bone density, vitamin D deficiency, secondary hyperparathyroidism, abnormal glucose metabolism, dyslipidaemia, gynecomastia, and hypogonadism [56,57,58]. 

Available evidence on the effects of TKIs on adrenal function is limited. In the preclinical phase of sunitinib development, adrenocortical hypertrophy, inflammation, congestion, haemorrhage, or necrosis were detected in rats and monkeys after exposure to the drug [59]. In contrast, no evidence of adrenal haemorrhage or necrosis was found in 336 patients who underwent a CT scan/MRI following the assumption of one or more cycles of sunitinib. Moreover, when ACTH stimulation testing was performed in approximately 400 patients with normal baseline ACTH across multiple clinical trials of sunitinib, only one patient showed abnormal test results during treatment. None of these patients were reported to have clinical evidence of AI. Despite these data, the Food and Drug Administration cautions that subclinical adrenal toxicity may be unmasked by physiologic stress in patients taking sunitinib [59]. Therefore, monitoring for AI is recommended in those patients who experience stress such as surgery, severe infection, or trauma [59].

A high prevalence (48% of cases) of subclinical hypocortisolism was shown among 25 patients affected by chronic myelogenous leukaemia treated with imatinib mesylate [60]. Another TKI tested for potential adrenal toxicity is vandetanib, either as a single agent or part of a combination regimen [61,62]. When the basal and stimulated adrenal function was assessed in 12 patients affected by advanced radioiodine refractory differentiated or medullary thyroid cancer who received lenvatinib and vandetanib, respectively, a gradual ACTH increase with normal cortisol levels was registered in 10 patients complaining of fatigue. Moreover, a cortisol response upon ACTH stimulation was detected in 6 of these patients, suggesting a PAI diagnosis. Renin and aldosterone were not evaluated. Patients diagnosed with PAI were treated with cortisone acetate replacement therapy, and fatigue improved [62]. 

Recently, PAI induced by pazopanib has been reported in a patient affected by metastatic kidney cancer [63]. No specific measure aimed at monitoring and preventing the risk of AI are cautioned for patients under these TKIs.

## 9. Adrenal Enzyme Inhibitors-Induced Adrenal Insufficiency

Abiraterone acetate is a noncytotoxic drug approved for the treatment of metastatic prostate cancer. As an inhibitor of 17α-hydroxylase and C17,20-lyase (CYP17), abiraterone blocks androgen synthesis and glucocorticoid production. Decreased cortisol levels result in an increased ACTH release, which can lead to increased mineralocorticoid levels. Coadministration of abiraterone and glucocorticoids has been demonstrated as effective in reducing mineralocorticoid excess. However, adequate replacement of physiologic glucocorticoids, especially in times of acute stress, remains to be well-defined [64]. Except for clinical surveillance, measures to monitor the risk of AI are not cautioned for patients under abiraterone.

## 10. Treatment of Primary Adrenal Insufficiency Induced by Anticancer Drugs

As glucocorticoids and mineralocorticoids are lacking in patients with PAI, the replacement of both hormones is required (together with salt intake if needed). By contrast, cases of ACTH deficiency due to pituitary or hypothalamic dysfunction (e.g., after treatment with steroids) usually require only glucocorticoid replacement. Patients with PAI or ACTH deficit also have androgen deficiency, but androgen replacement is still controversial, as the derived benefits remain to be confirmed [65,66,67]. However, treating patients diagnosed with PAI induced by anticancer drugs may be difficult because attention should be paid to both the replacement therapy and the management of the causative drug(s) that rarely can be substituted (Figure 2). 

A key aspect of the replacement therapy is the administration of hydrocortisone (10–12 mg/m2/day) or cortisone acetate (20–35 mg daily). Moreover, a daily titration dose in 2 or 3 doses is required to mimic endogenous cortisol rhythm [33]. Two or three divided oral doses per day are recommended, with the highest dose assumed in the morning at awakening. In the two-dose regimen, the second next should be given in the early afternoon, about two hours after lunch; two doses should be administered at lunch and afternoon in the three-dose regimen. If available, modified-release formulations of hydrocortisone in clinical could allow a single administration. In the case of aldosterone deficiency, mineralocorticoid replacement with fludrocortisone is advised with a starting dose in adults of 0.05–0.1 mg to reduce at the onset of arterial hypertension [21].

Patients diagnosed with mitotane-induced PAI need specific prescriptions. Indeed, the replacement therapy requires a double dose of glucocorticoids than generally used in other forms of PAI (40–50 mg hydrocortisone or 75 mg cortisone acetate). Notably, modified-release hydrocortisone is not suggested, as it seemed to reach lower total and free cortisol levels than immediate-release hydrocortisone at the same dosage. This may not assure the physiological morning free cortisol peak [68]. Notably, inadequate treatment of AI may worsen mitotane-related toxicity, particularly in terms of gastrointestinal AEs. In turn, this may reduce tolerance to mitotane, leading to early discontinuation of the drug [69]. In these patients, the supplementation with mineralocorticoids is not always required as the zona glomerulosa of the adrenal glands is less vulnerable to mitotane’s toxic effect. When clinical and biochemical signs of aldosterone deficiency appear, starting fludrocortisone (0.1 to 0.3 mg daily) is suggested, considering that the metabolism of fludrocortisone is increased by mitotane [70]. 

## 11. Management of the Anticancer Drug Causing Primary Adrenal Insufficiency

Once ICI-induced PAI is diagnosed, ICI(s) should be provisionally held and managed according to the level of adrenal toxicity. Current guidelines on the management of ICI-induced endocrine toxicity provide detailed approaches, including ICI-induced PAI (Table 4). In general, once the patient is stabilized and the appropriate replacement endocrine therapy is instituted, immunotherapy may continue in the absence of other severe or life-threatening irAEs (Figure 2). Notably, as adrenal function infrequently recovers following ICI-induced PAI, physiologic hormone replacement will likely be required indefinitely (typically life-long). Moreover, as the goal for physiologic steroid replacement is to identify the lowest steroid dose needed to prevent symptoms of AI, patients who experienced ICI-induced PAI should be monitored by an endocrinologist. It is vital to provide patient education regarding stress doses of hydrocortisone in infection, trauma, or other medical or surgical events. Moreover, patients should wear a medical alert bracelet or bring with them an emergency identification card [35,46,49,50,52,53,54,55].

In patients under TKIs, no specific measures regarding the management of the causative drug are currently advised. Modification of drug dosing can be based on the level of the reported toxicity through a collaboration between oncologists and endocinologists (Figure 2). Similar recommendations are advisable in the sporadic occurrence of PAI induced by other anticancer drugs.

## 12. Conclusions

PAI induced by anticancer drugs is a rare occurrence. However, with the expanding use of ICIs, an increasing number of patients affected by ICI-induced PAI are expected. Oncologists should be aware of the risk of PAI in patients on ICIs (and other anticancer agents) and the related clinical presentations. This will enable the early diagnose of PAI and the timely involvement of endocrinologists in the management of the patients to provide them with the appropriate treatment. Patients affected by any form of adrenal insufficiency, including those with anticancer drug induced PAI, and their partner/family members are strongly recommended to be well aware of the related alarm signs and symptoms (including nausea, vomiting, abdominal pain, hypoglycemia, hypotension, weight loss, etc.) and be trained to act appropriately at their onset. Importantly, patients should be equipped with an emergency identification card and/or a medical alert bracelet, as well as a kit for self-treatment of acute AI (adrenal crisis) (including appropriate needles, syringes, and water for injection reconstitution) available at all times [21,46,53,54,55]. Moreover, they must know the correct adjustment of glucocorticoid replacement dose when specific circumstances occur. Patients need to double the usual oral glucocorticoid dose in case of fever or illness requiring bed rest, when antibiotics are necessary to treat an infection, when approaching minor outpatient procedures (e.g., dental work). Patients with severe illness, trauma, persistent vomiting, or fasting for a procedure (e.g., colonoscopy), should have an extra supply of IV/IM hydrocortisone. Similarly, those undergoing elective surgery should receive a stress dose of hydrocortisone 100 mg IV before induction of anaesthesia, during and/or after the operation (depending on the type, severity, and duration of surgery). Additionally, patients and their partner/family members should have an accessible telephone number of the reference care team to call in case of any doubt about the management of replacement therapy and adverse events [21,46,53,54,55]. Finally, large collaborative trials are needed to develop appropriate tests to better assess the personal risk of drug induced PAI and improve its early diagnosis, not only in cancer patients, but also in those affected by other forms of PAI.

## Figures and Tables

**Table 1:** Aetiology of primary adrenal insufficiency in adults.

Causes	Pathogenesis	Frequency
Autoimmunity	T and B cell autoimmunity against adrenocortical cells	70–90%
Infectious diseases	Mycobacteria, bacteria (e.g., Neisseria meningitidis, Haemophilus influenzae, Pseudomonas aeruginosa), viruses (e.g., human immunodeficiency virus, herpes simplex, cytomegalovirus) or fungi (e.g., Pneumocystis jirovecii)	10–30%
Tumour	Primary tumour (bilateral), metastases (bilateral), adrenal lymphoma (bilateral)	
Bleeding	Anti-phospholipid syndrome, anticoagulant therapy, disseminated intravascular coagulation	
Surgery	Bilateral adrenalectomy	
Infiltrative	Amyloidosis, hemochromatosis, histiocytosis	
Genetic	Congenital adrenal hyperplasia, congenital lipoid adrenal hyperplasia, adrenoleukodystrophy (X- linked), adrenal hypoplasia congenita, autoimmune polyglandular syndrome type 1	
Drugs	Enzyme inhibition (ketoconazole, fluconazole, itraconazole, etomidate, aminoglutethimide, metyrapone, rifampicin, phenytoin, phenobarbital, trilostane, osilodrostat etomidate, suramine, mifepristone, tramadol, abiraterone acetate) Adrenolytic effect and increased cortisol metabolism (mitotane, tramadol) Autoimmunity/inflammation (immune checkpoint inhibitors) Uncertain/unknown (tyrosine kinase inhibitors)	

**Table 2:** Laboratory tests in primary adrenal insufficiency.

Assessment	Results
Plasma ACTH (early morning)	Normal or elevated
Serum cortisol level	Inappropriately reduced (Attention to cortisol binding globulin or albumin alterations, such as in cirrhosis, nephrotic syndrome, or individuals on oral oestrogen treatment)
Cosyntropin stimulation test (250 mcg i.v)	Peak cortisol levels <18 mcg/dL (<500 nmol/l)
Renin or plasma renin activity	Increased
Aldosterone	Reduced

**Table 3:** Suggested stages of adrenal insufficiency.

Stage	Laboratory Findings
I	High plasma renin activity and normal or low serum aldosterone
II	Impaired serum cortisol response to ACTH stimulation
III	Increased morning plasma ACTH with normal serum cortisol
IV	Low morning serum cortisol and overt clinical adrenal insufficiency

**Table 4:** Current guidelines of American Society of Clinical Oncology on the management of PAI and ICI(s) in ICI-induced PAI according to the CTC-AE grading system [50].

	PAI	ICI(s)
Grade 1 (Asymptomatic or mild symptoms)	Endocrine consultation Prednisone (5 to 10 mg daily) or hydrocortisone (10 to 20 mg orally in the morning, 5 to 10 mg orally in early afternoon) Fludrocortisone (0.1 mg/d) Titrate dose up or down as symptoms dictate	May hold ICIs until the patient is stabilized on replacement hormone
Grade 2 (Moderate symptoms)	Endocrine consultation Treatment at two to three times maintenance Should taper stress-dose corticosteroids down to maintenance doses over 5 to 10 days. Should offer maintenance therapy as in grade 1	May hold ICIs until the patient is stabilized on replacement hormone
Grade 3 (Severe symptoms) Or Grade 4 (life-threatening)	Endocrine consultation Hospitalization for normal saline (at least 2 L) and IV stress-dose corticosteroids on presentation (Hydrocortisone 100 mg or dexamethasone 4 mg) Taper stress-dose corticosteroids down to maintenance doses over 7–14 days after discharge. Should offer maintenance therapy as in grade 1	Should hold ICIs until the patient is stabilized on replacement hormone
